# Which risk factor best predicts coronary artery disease using artificial neural network method?

**DOI:** 10.1186/s12911-024-02442-1

**Published:** 2024-02-14

**Authors:** Nahid Azdaki, Fatemeh Salmani, Toba Kazemi, Neda Partovi, Saeede Khosravi Bizhaem, Masomeh Noori Moghadam, Yoones Moniri, Ehsan Zarepur, Noushin Mohammadifard, Hassan Alikhasi, Fatemeh Nouri, Nizal Sarrafzadegan, Seyyed Ali Moezi, Mohammad Reza Khazdair

**Affiliations:** 1 Cardiovascular Diseases Research Center, Birjand University of Medical Sciences, Birjand, Iran; 2 Clinical Research Development Unit, Razi Hospital, Birjand University of Medical Sciences, Birjand, Iran; 3 Department of Epidemiology and Biostatistics, School of Health, Social Determinants of Health Research Center, Birjand University of Medical Sciences, Birjand, Iran; 4 Interventional Cardiology Research Center, Cardiovascular Research Institute, Isfahan University of Medical Sciences, Isfahan, Iran; 5 Pediatric Cardiovascular Research Center, Cardiovascular Research Institute, Isfahan University of Medical Sciences, Isfahan, Iran; 6 Heart Failure Research Center, Isfahan Cardiovascular Research Institute, Isfahan University of Medical Sciences, Isfahan, Iran; 7 Hypertension Research Center, Cardiovascular Research Institute, Isfahan University of Medical Sciences, Isfahan, Iran; 8 Isfahan Cardiovascular Research Center, Cardiovascular Research Institute, Isfahan University of Medical Sciences, Isfahan, Iran

**Keywords:** Coronary artery disease, Artificial neural network, Data mining, Risk factors

## Abstract

**Background:**

Coronary artery disease (CAD) is recognized as the leading cause of death worldwide. This study analyses CAD risk factors using an artificial neural network (ANN) to predict CAD.

**Methods:**

The research data were obtained from a multi-center study, namely the Iran-premature coronary artery disease (I-PAD). The current study used the medical records of 415 patients with CAD hospitalized in Razi Hospital, Birjand, Iran, between May 2016 and June 2019. A total of 43 variables that affect CAD were selected, and the relevant data was extracted. Once the data were cleaned and normalized, they were imported into SPSS (V26) for analysis. The present study used the ANN technique.

**Results:**

The study revealed that 48% of the study population had a history of CAD, including 9.4% with premature CAD and 38.8% with CAD. The variables of age, sex, occupation, smoking, opium use, pesticide exposure, anxiety, sexual activity, and high fasting blood sugar were found to be significantly different among the three groups of CAD, premature CAD, and non-CAD individuals. The neural network achieved success with five hidden fitted layers and an accuracy of 81% in non-CAD diagnosis, 79% in premature diagnosis, and 78% in CAD diagnosis. Anxiety, acceptance, eduction and gender were the four most important factors in the ANN model.

**Conclusions:**

The current study shows that anxiety is a high-prevalence risk factor for CAD in the hospitalized population. There is a need to implement measures to increase awareness about the psychological factors that can be managed in individuals at high risk for future CAD.

## Background

Cardiovascular diseases (CVDs) are prevalent conditions that affect the heart and vessels, resulting in approximately 31% of all global deaths [1]. CVDs encompass a range of diseases, such as acute coronary syndrome, stroke, heart failure, coronary heart disease, cardiomyopathies, and peripheral vascular diseases [2]. Diabetes, dyslipidemias, obesity, hypertension, low or lack of physical activity, and smoking are cardiovascular-related risk factors [3].

The incidence of CVD may increase due to changes in lifestyles, such as physical inactivity, smoking, drug intake, and an increase in the prevalence of type 2 diabetes mellitus [4]. In addition to the primary risk factors, infection and chronic diseases have recently been considered to be additional risk factors for CVDs [5]. The burden of CVDs in Iran has been projected to rise sharply from 2005 to 2025, primarily due to the country’s aging population. The DALY related to CVDs is predicted to increase by more than two-fold in 2025 compared to 2005 [6]. Identifying and preventing the mentioned risk factors can reduce the prevalence of CVDs. In addition to lifestyle modifications, lipid-lowering drugs, antihypertensives, and antiplatelet and anticoagulant medications are the main prescriptions for preventing and treating CVDs [7].

Data mining, or machine learning, is a process that involves identifying anomalies, patterns, and correlations within large datasets to make predictions that have broad applications in medicine [8]. Various data mining algorithms, such as clustering, decision trees, and neural networks, have been utilized to predict CVDs [9, 10]. Machine learning and data mining methods can be highly beneficial in clinical decision-making because they provide significant decision-making power.

The artificial neural network (ANN) is favored among data mining algorithms due to its simplicity, high speed, and ability to solve complex relationships between variables [11]. The ANN is one of the machine learning techniques with the highest prediction accuracy. This approach has been implemented in numerous studies to determine predictors of Coronary Artery Disease (CAD) [12, 13]. Although, association between coronary heart disease (CHD) risk factors including; age, gender, lipid profle, arterial hypertension, fasting plasma glucose, smoking status, obesity and serum interferon in related CHD were assessed using ANN previously, but the association between demographic characteristic, lifestyle risk factors such as, stress and anxiety as well as clinical risk factors in relation to CAD were not assessed. This study uses the ANN to compare and predict CVD risk factors in a selected hospital.

## Methods

### Design and study population

The present study is a subsection of a multi-center study in Iran, namely the Iran-premature coronary artery disease (I-PAD), which has been previously described in depth [14]. This study included angiographic patients with occlusion of at least one coronary artery ≥ 75% or left main coronary ≥ 50%, as well as control subjects with normal coronary arteries. The participants were selected from Razi Hospital in Birjand, Iran, between 2016 and 2019. The patients undergoing coronary angiography were limited to men and women aged ≤ 60 or ≤ 70 years, respectively. The criteria for premature CAD patients consisted of age ≤ 45 or ≤ 55 years for men and women, respectively. Patients who had previously undergone coronary artery bypass grafting, balloon angioplasty, or percutaneous coronary intervention were excluded from the study. This study determined 500 patients undergoing coronary angiography in Birjand between 2016 and 2019 as the sample size. Patients were searched through the Isfahan angiographic data registration system, and if they met the inclusion criteria, they were contacted by interviewers and invited to participate in the study. Additionally, eligible angiographic patients hospitalized in the ward were selected for questioning. Among the 415 individuals included in the study, 54.9% (228) were female. The mean age of the participants was 54.10 ± 7.68 years. Among the participants, 200 individuals (48.2%) exhibited CVD, characterized by the presence of at least one vascular stenosis exceeding 50% in the left main artery and 70% in other vessels. The remaining 215 participants (51.8%) did not show signs of CAD. Within this group, 39 individuals (9.4%) had premature CAD, while 161 individuals (38.8%) had CAD.

The study received approval from the Ethics Committee of Isfahan University of Medical Sciences (IR.MUI.REC.1396.2.055).

### Data collection

The data collected pertained to age, sex, religion, ethnicity, education, occupation, economic status of the family, history of drug use, smoking, and alcohol consumption, as well as personal and family history of CVD and medications. Behavioral patterns (anxiety, depression, and coping with stress) were collected using the Hospital Anxiety and Depression Scale, sleep quality using the Pittsburgh Sleep Quality Index, physical activity using the International Physical Activity Questionnaire, and dietary patterns using the Food Frequency Questionnaire. Then, according to standard protocols, height, waist circumference, hip circumference, neck circumference, and thighs were measured for each patient. The methodology paper of this study provides a thorough description of the study’s methodology [14]. Each group’s sample size was calculated using a = 0.05, a power of 0.8, and an odds ratio of 1.30. Individuals were recruited through convenience sampling in hospitals across the country [14].

The following instruments were used in the current study: Physical Activity Questionnaire (20 items), Stress Coping Questionnaire (23 items), Depression Questionnaire (17 items), Environmental Questionnaire, Sensitivity Questionnaire, Oral and Dental Questionnaire, Sleep Quality Questionnaire (17 items), Sexual Activity Questionnaire (10 items), Drug Use Questionnaire, Cigarettes and Alcohol Questionnaire, Biography Questionnaire that includes personal or familial history of the disease, Medications Questionnaire, Family Economic Status Questionnaire (12 items), and Nutrition Questionnaire (112 items). Moreover, the Physical Examination Questionnaire was administered, which includes patients’ vital signs (pulse, systolic blood pressure (SBP), and diastolic blood pressure (DBP). These parameters were measured twice, 15 min apart [14].

General tests were taken and recorded after 12 h of fasting. They comprised total cholesterol (Chol), triglyceride (TG), high-density lipoprotein cholesterol (HDL-c), low-density lipoprotein cholesterol (LDLc), and fasting blood sugar (FBS).

Diabetes Mellitus (DM) defined FBS ≥ 126 mg/dl or history of diabetes, and DLP defined as history of DLP or one of lipid profile was abnormal. Lipid profile including TC, TG, LDL, HDL and FBS standardized according to the defined values. Based on the standards, the normal and abnormal values of the laboratory test were considered as follows: TG ≥ 150 mg/dl, Chol ≥ 240 mg/dl, LDL ≥ 100 mg/dl and HDL < 40 mg/dl in males and < 50 mg/dl for females considered abnormal.

### Statistical analysis

Inspired by the function of the human brain, the ANN refers to a family of models with a large parametric space and a flexible structure. A neural network, in fact, is a parallel distribution processor that aims to store experimental knowledge and make it usable [15]. Any neural network that includes a hidden layer has the potential to be a reliable predictor, provided that there is an adequate number of hidden neurons [16]. However, there is no theoretical consensus on finding the optimal number of hidden neurons and functions between layers [17]. One type of ANN is the feed-forward network, in which the path always moves forward and does not return to the neurons of the previous layer.

According to Akella and Akella (2021), the neural network (NN) is the best method for predicting and diagnosing CAD. The reported accuracy of the NN is 93%, with a specificity of 0.93 and a sensitivity of 0.89 [18]. The steps of the ANN in our study include data preparation, model creation, assessment, and interpretation. During the data preprocessing step, several preprocessing approaches were utilized on the dataset to ensure the integrity and suitability of the data for analysis using ANN. One task involved in the data cleaning process was identifying outliers and data inconsistencies to maintain the integrity of the dataset [19].

In order to address issues related to the different magnitudes of variables, a standardization process was implemented during the scaling stage. This process aimed to establish a uniform scale for continuous variables so as to ensure consistency. The variance inflation factor was used to identify the presence of multicollinearity among variables, with a threshold of 5 being regarded as an indicator of multicollinearity [20].

The dataset was randomized to create three distinct subgroups. The subsets consisted of a training set, which comprised 70% of the data and was utilized for model training, a testing set that accounted for 20% of the data, and a validation set that constituted 10% of the data [21]. The architectural design incorporated the input layer, which was responsible for including the predictor variables relevant to the study. The optimal number of layers and neurons was determined through a methodical approach that involved conducting experiments and refining hyperparameters. The output layer was implemented using sigmoid activation functions.

The development of the model’s progression:

The training protocol for the ANN model included the following phases:

The training dataset was processed using a feed-forward network [22] in order to generate predictions. The convergence criteria for training involved determining when to stop training based on specific parameters. These parameters included reaching a maximum number of epochs or achieving adequate performance on the validation set. A minimum threshold of 0.001 was established for the relative change in the training error ratio. The criterion mentioned above suggests that it is advisable to stop the training process when the accuracy level exceeds a specific threshold at a particular training stage, also known as early quitting. Additionally, examining concealed layers involved analyzing a wide range of hidden levels, spanning from 1 to 50 [23].

The evaluation of the model:

The performance of the model was evaluated across several classes using Receiver Operating Characteristic (ROC) curves. The model’s performance was assessed by quantifying the area under the ROC curve, commonly referred to as the AUC.

The data analysis in this study was conducted using the SPSS 26 program. The mean and standard deviation were used to describe quantitative data, while counts and percentages were used to represent qualitative factors. An independent t-test was used to evaluate the mean differences of quantitative variables between the premature CAD and non-CAD groups. An analysis of variance (ANOVA) was used to assess the differences in means between premature CAD, early CAD, and non-CAD groups. The chi-square test was employed to analyze qualitative factors across study groups. The exact Fisher’s test was used when the expected frequency in more than 20% of the cells was less than 5. Logistic regression was applied to predict the odds ratio of study variables for the occurrence of premature CAD. The assumption of linearity in logistic regression was tested using the Box-Tidwell test [24]. Additionally, ordinal logistic regression was used to model the status of premature CAD at three levels: premature, early CAD, and non-CAD.

## Results

The demographic characteristics, medical history, and lifestyle of the premature CAD and non-CAD groups are presented in Table 1. Gender, education, occupation, smoking, opium use, diabetes mellitus, high blood sugar, high LDL, and history of diabetes mellitus differed significantly between the two groups (*p* < 0.05 to *p* < 0.001). Lifestyle risk factors, such as acceptance, exposure to poisons, anxiety, and seasonal allergies and sex activity showed significant differences between patients with premature CAD and non-CAD patients (*p* < 0.01 and *p* < 0.001, respectively). The two groups had no statistically significant difference for the other variables (Table 1). The analysis was conducted using the simple logistic regression method.

**Table 1:** Crude CAD odds ratio and demographic, lifestyle, and clinical risk factors by logistic regression in patients without CAD and premature CAD

		Without CAD (*n* = 215)	Premature CAD (*n* = 200)	*P*-Value	Crude OR	95% CI OR
*Demographic characteristics*						
Age		53.61 ± 8.03	54.62 ± 7.27	0.185	1.02	0.99–1.04
BMI		27.55 ± 5.21	26.94 ± 5.46	0.241	0.98	0.94–1.02
WC		93.12 ± 11.98	93.76 ± 10.42	0.59	1	0.98–1.02
SBP		120.87 ± 16.03	123.87 ± 17.51	0.068	1.01	0.10–1.02
DBP		78.81 ± 9.88	79.51 ± 11.01	0.497	1.35	0.77–2.37
Sex (female) n (%)		155 (72)	73 (36.5)	< 0.001	0.22	0.15–0.34
Education n (%)	No formal education	60 (27.9)	50 (25)	0.053	0.48	0.25–0.92
	Primary school	75 (34.9)	52 (26)		0.40	0.21–0.76
	Gaudiness school	26 (12.1)	23 (11.5)		0.51	0.24–1.10
	High school	32 (14.9)	37 (18.5)		0.67	0.33–1.36
	BS and MS	22 (10.2)	38 (19)		Reference	
Job n (%)	Employee	43 (20)	49 (24.5)	< 0.001	0.41	0.19–0.90
	Self-employee	31 (14.4)	52 (26)		0.61	0.28–1.35
	housewife	129 (60)	66 (33)		0.19	0.09–0.38
	Jobless and retired	12 (5.6)	33 (16.5)		Reference	
Marital status n (%)	married	192 (89.3)	187 (93.5)	0.129	Reference	
	single	23 (10.7)	13 (6.5)		1.72	0.85–3.50
Family Economic		6.55 ± 2.29	6.75 ± 2.63	0.419	1.03	0.96–1.12
Smoke n (%)		28 (13)	73 (36.5)	< 0.001	3.84	2.35–6.27
Opium n (%)		32 (16.3)	75(37.7)	< 0.001	3.11	1.96–4.94
*Clinical Risk factors*						
DM n (%)		56 (26.2)	73 (36.7)	0.021	1.64	1.08–2.49
High FBS n (%)		35 (39.3)	54 (27.1)	0.008	1.91	1.18–3.07
High LDL n (%)		29 (13.5)	15 (7.5)	0.05	0.52	0.27–1.01
High Chol n (%)		10 (4.7)	6 (3)	0.388	0.64	0.23–1.79
High TG n (%)		28 (13)	33 (16.6)	0.307	1.33	0.77–2.29
Low HDL n (%)		106 (49.3)	98 (49.2)	0.991	0.10	0.68–1.47
DLP n (%)		165 (76.7)	148 (74.4)	0.574	0.88	0.56–1.38
History DLP		84(39.1)	95(47.5)	0.09	1.41	0.96–2.08
History DM		49(22.8)	63(31.7)	0.047	1.57	1.01–2.43
History HTN		82(38.1)	85(42.5)	0.37	1.2	0.81–1.78
*Life Style Risk factors*						
Stress	SSS	10.01 ± 3.52	10.62 ± 3.04	0.061	1.05	0.99–1.12
	PE	9.00 ± 0.2.10	9.23 ± 2.17	0.273	1.05	0.96–1.15
	PG	6.55 ± 1.44	6.81 ± 1.27	0.061	1.15	0.99–1.32
	Acceptance	3.06 ± 0.93	3.33 ± 0.79	0.002	1.43	1.14–1.8
	Avoidance	3.21 ± 1.45	3.29 ± 1.60	0.599	1.03	0.91–1.18
Anxiety		5.79 ± 2.59	5.11 ± 2.53	0.007	0.90	0.83–0.97
Depression		8.93 ± 2.25	8.84 ± 2.26	0.68	0.98	0.90–1.07
Poison Exposure n (%)		40 (18.6)	64 (32)	0.002	2.06	1.31–3.24
Use Mobile n (%)		185 (86)	180 (90)	0.216	1.46	0.80–2.66
Seasonal allergy		59(27.4)	28(14)	0.001	0.84	0.75–0.94
Medical allergy		18(8.4)	11(5.5)	0.255	0.90	0.72–1.12
Food allergies		11(5.1)	7(3.5)	0.417	0.91	0.73–1.13
Skin allergy		28(13)	17(8.5)	0.126	0.84	0.70-1.00
Sleep quality		7.41 ± 1.58	7.52 ± 1.66	0.53	1.04	0.92–1.17
Sex activity		6.27 ± 1.70	6.88 ± 1.72	< 0.001	1.24	1.10–1.39

BMI: Body Mass Index, WC: Waist circumstance, SBP: Systolic blood pressure, DBP: Diastolic blood pressure, SSS: Seeking Social Support, PE: Problem Engagement, PG: Positive Growth, FBS: Fasting blood sugar, DM: Diabetes mellitus, DLP: Dyslipidemia, HTN: Hypertension

The underlying factors were also compared between the three groups of non-CAD, early CAD, and premature CAD patients. The variables of age, sex (female), occupation, smoking, opium use, exposure to poisons, anxiety, seasonal allergy, sexual activity, and high fasting blood sugar were significantly different in the three groups (*p* < 0.05 to *p* < 0.001) (Table 2). The analysis was conducted using the simple ordinal logistic regression method.

**Table 2:** Crude CAD odds ratio and demographic, lifestyle, and clinical risk factors by ordinal logistic regression in patients without CAD, early CAD, and premature CAD

		Without CAD (*n* = 215)	Early CAD (*n* = 39)	Premature CAD (*n* = 161)	*P*-Value	Crude OR	95% CI OR
*Demographic characteristics*							
Age		53.61 ± 8.03	45.59 ± 5.57	56.80 ± 5.80	< 0.001	1.04	1.02–1.07
BMI		27.55 ± 5.21	26.75 ± 4.21	26.99 ± 5.73	0.488	0.98	0.95–1.02
WC		93.11 ± 11.98	93.18 ± 8.85	93.90 ± 10.78	0.78	1	0.98–1.02
SBP		120.87 ± 16.03	123.96 ± 16.64	123.87 ± 17.77	0.190	1.01	0.99–1.02
DBP		78.81 ± 9.88	81.82 ± 10.79	78.94 ± 111.02	0.242	1.00	0.98–1.02
Sex (female) n (%)		155 (72.1)	19 (48.7)	54 (33.5)	< 0.001	0.23	0.153–0.337
Education n (%)	No formal education	60 (27.9)	6 (15.4)	44 (27.3)	0.088	0.59	0.32–1.08
	Primary school	75 (34.9)	11 (28.2)	41 (25.5)		0.46	0.25–0.83
	Gaudiness school	26 (12.1)	3 (7.7)	20 (12.4)		0.62	0.30–1.28
	High school	32 (14.9)	10 (25.6)	27 (16.8)		0.69	0.36–1.35
	BS and MS	22 (10.2)	9 (23.1)	29 (18)		Reference	
Job n (%)	Employee	43 (20)	11 (28.2)	38 (23.6)	< 0.001	0.35	0.17–0.72
	Self-employee	31 (14.4)	8 (20.5)	44 (27.3)		0.53	0.25–1.12
	housewife	129 (60)	18 (46.2)	48 (29.8)		0.16	0.08–0.31
	Jobless and retired	12 (5.6)	2 (5.1)	31 (19.3)		Reference	
Marital status n (%)	married	192 (89.3)	37 (94.9)	150 (93.2)	0.351	Reference	
	single	23 (10.7)	2 (5.1)	11 (6.8)		1	0.305–1.215
Family Economic		6.55 ± 2.28	6.74 ± 2.21	6.75 ± 2.72	0.33	1.03	0.95–1.11
Smoke n (%)		28 (13)	12 (30.8)	61 (37.9)	< 0.001	3.48	2.21–5.47
Opium n (%)		23 **(**10.7)	10 (25.6)	44 (27.3)	< 0.001	4.34	1.88–9.99
Clinical Risk factors							
DM n (%)		56 (26.2)	13 (33.3)	60 (37.5)	0.062	0.62	0.414–0.924
High FBS n (%)		35 (16.4)	11 (28.2)	43 (26.9)	0.028	0.565	0.359–0.889
High LDL n (%)		29 (13.5)	3 (7.7)	12 (7.5)	0.169	1.87	0.984–3.557
High Chol n (%)		10 (4.7)	1 (2.6)	5 (3.1)	0.802	1.51	0.553–4.133
High TG n (%)		28 (13)	8 (20.5)	25 (15.6)	0.441	0.82	0.483–1.374
Low HDL n (%)		106 (49.3)	24 (61.5)	74 (46.3)	0.231	1.09	0.751–1.581
DLP n (%)		165 (76.7)	34 (87.2)	114 (71.3)	0.10	1.28	0.829–1.966
History DLP n (%)		84 (39.1)	21 (53.8)	74(46)	0.15	1.31	0.90–1.92
History DM n (%)		49(22.8)	12(30.8)	51(31.9)	0.13	1.52	1-2.32
History HTN n (%)		82(38.1)	14(35.9)	71(44.1)	0.42	1.24	0.85–1.81
Life Style Risk factors							
Stress	SSS	10.01 ± 3.52	10.72 ± 3.46	10.59 ± 2.94	0.173	1.05	0.99–1.11
	PE	9.00 ± 2.10	9.51 ± 2.06	9.16 ± 2.19	0.365	1.03	0.95–1.13
	PG	6.56 ± 1.44	6.56 ± 1.50	6.87 ± 1.21	0.079	1.16	1.00-1.33
	Acceptance	3.05 ± 0.93	3.23 ± 0.87	3.36 ± 0.77	0.005	1.43	1.15–1.79
	Avoidance	3.22 ± 1.45	2.84 ± 1.68	3.41 ± 1.52	0.098	1.06	0.94–1.20
Anxiety		5.79 ± 2.59	4.89 ± 2.37	5.16 ± 2.57	0.023	0.91	0.84–0.98
Depression		8.93 ± 2.25	8.85 ± 2.26	8.88 ± 2.25	0.89	0.98	0.90–1.07
Exposure to Poisons		40 (18.6)	8 (20.5)	56 (34.8)	0.001	2.17	1.41–3.35
Use Mobile		185 (86)	34 (87.2)	146 (90.7)	0.381	1.49	0.82–2.68
Seasonal allergy		59(27.4)	7(17.9)	21(13)	0.003	0.84	0.75–0.94
Medical allergy		18(8.4)	3(7.7)	8(5)	0.441	0.90	0.72–1.11
Food allergies		11(5.1)	1(2.6)	6(3.7)	0.683	0.92	0.74–1.13
Skin allergy		28(13)	3(7.7)	14(8.7)	0.305	0.84	0.71-1.00
Sleep quality		7.41 ± 1.58	7.54 ± 1.94	7.51 ± 1.61	0.81	1.03	0.92–1.16
Sexual activists		6.26 ± 1.70	6.78 ± 2.12	6.90 ± 1.61	0.001	0.19	0.08–0.31

BMI: Body Mass Index, WC: Waist circumstance, SBP: Systolic blood pressure, DBP: Diastolic blood pressure, SSS: Seeking Social Support, PE: Problem Engagement, PG: Positive Growth, FBS: Fasting blood sugar, DM: Diabetes mellitus, DLP: Dyslipidemia, HTN: Hypertension

**Fig. 1:**
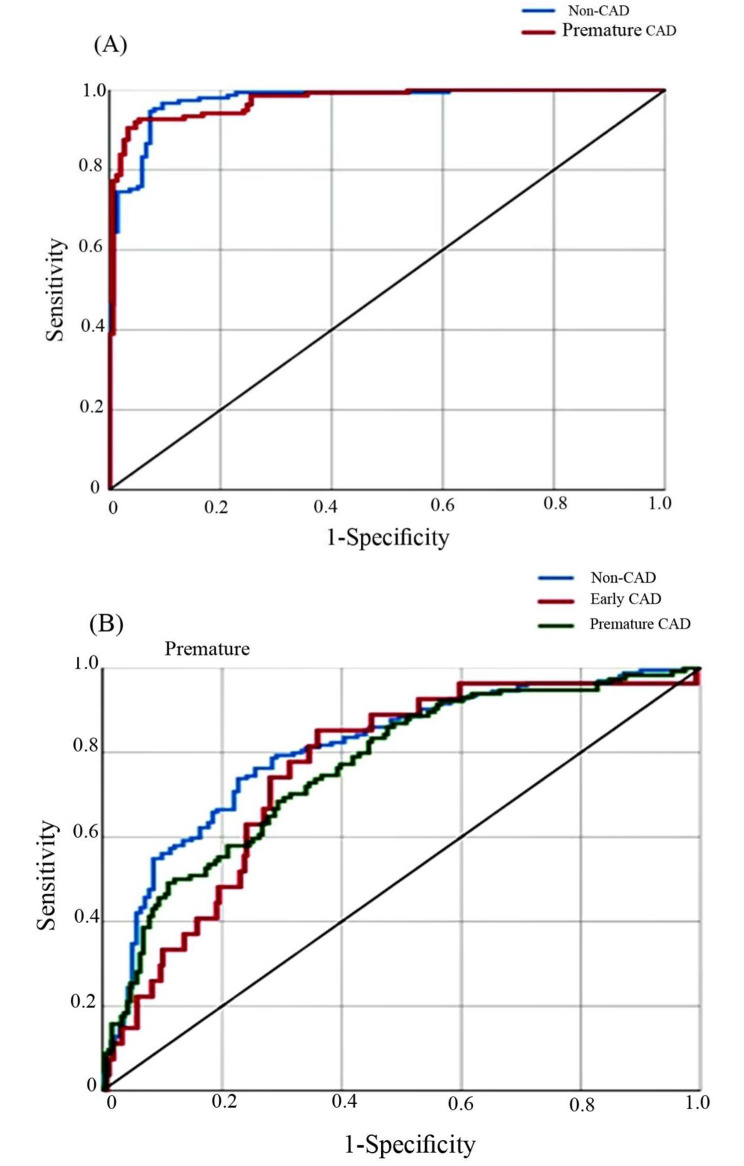
The model with 98% accuracy in diagnosing premature CAD and non-CAD patients (**A**), 81% accuracy in non-CAD, 79% in Early CAD, and 78% in premature CAD diagnosis (**B**)

The neural network method was used to investigate the simultaneous effect of input variables and eliminate any possible alignment effects between them. The efficiency of the models was calculated by considering the number of hidden layers and hidden nodes, and the results of the ROC curve analysis were presented. The neural network was fitted with seven hidden layers for the premature CAD/non-CAD output variable. The final model achieved a diagnostic accuracy of 98% in distinguishing between premature CAD and non-CAD patients (Fig. 1a).

Furthermore, the output variable proposed in the model (non-CAD, early CAD, premature CAD) was assessed using the same significant input variables listed in Table 2. The neural network achieved success with five hidden fitted layers and an accuracy of 81% in non-CAD diagnosis, 79% in early diagnosis, and 78% in premature CAD diagnosis (Fig. 1b).

**Fig. 2:**
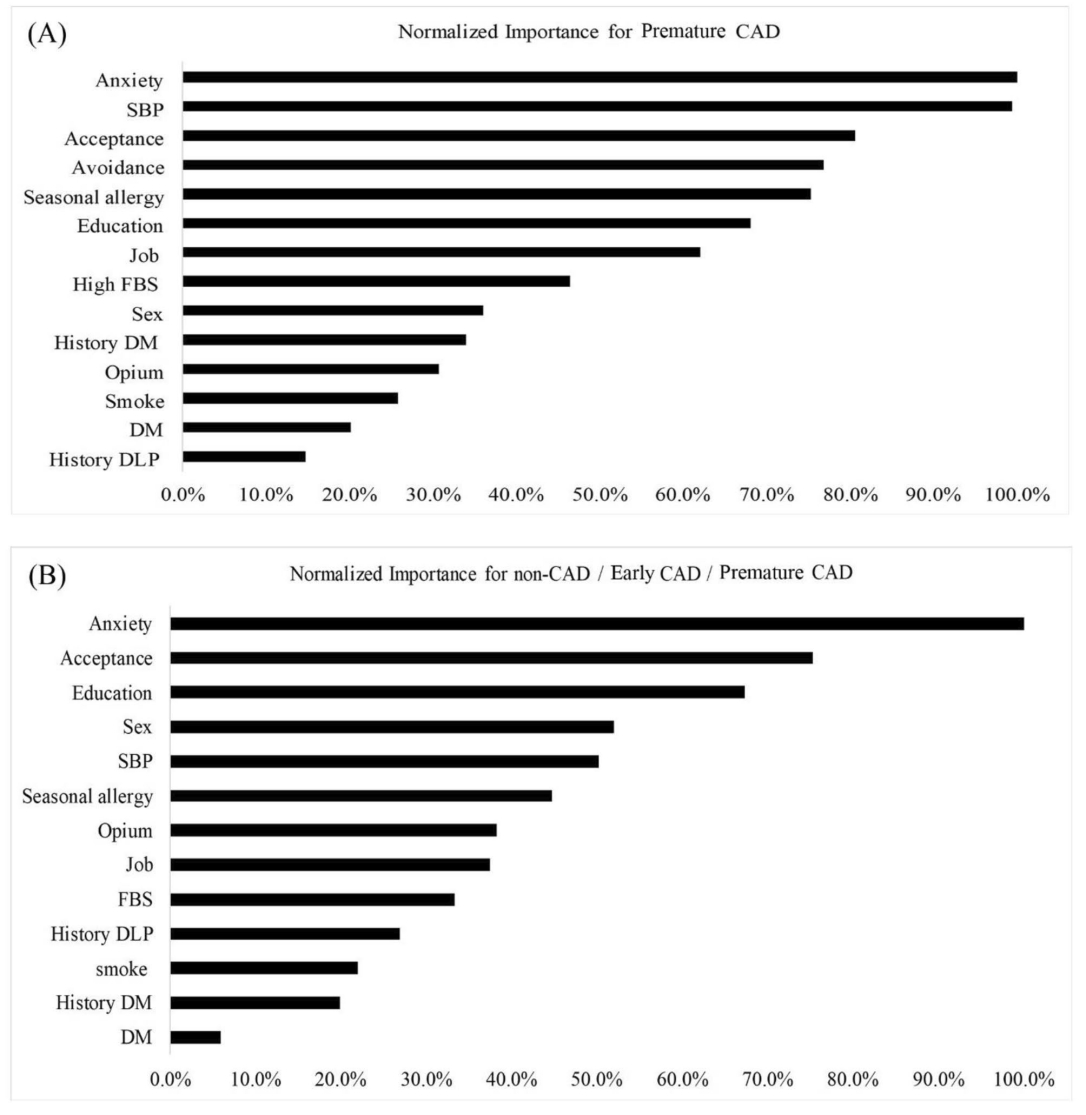
The importance of factors in the occurrence of premature CAD. **A**: Comparison between non-CAD / premature CAD groups. **B**: Comparison between non-CAD / Early CAD / premature CAD groups

Figure 2 displays the obtained ranking and level of importance of the risk factors. The most significant factors contributing to premature CAD were anxiety (100%), high blood pressure (99.4%), acceptance (80.5%), avoidance (76.9%), and seasonal allergies (75.3%). Low level of education (68.1%) and occupation (62%) were ranked next in terms of importance. Opium (30.7%) and smoking use (25.8%) were identified in the network with less than 50% prevalence (Fig. 2a).

According to the neural network model for output variables (non-CAD / early CAD / premature CAD), the most important factors identified were anxiety (100%), acceptance (75.3%), education (67.3%), gender (51.9%), and high blood pressure (50.2%). The importance rank of opium was 38.3% (Fig. 2b).

## Discussion

The results of the current study indicate that the important risk factors affecting the incidence of CAD are gender, age, education, occupation, smoking, opium use, pesticide exposure, history of dyslipidemia, history of diabetes, high blood sugar, acceptance, anxiety, seasonal allergy and sexual activity using the simple logistic regression method.

The incidence of the disease in the current study was found to be lower in women than in men. Additionally, it was observed that age and weight could potentially increase the risk of CAD. Previous research has also demonstrated that certain risk factors, including age, smoking, and sex (male), exhibit positive correlations with CAD [11, 25]. Furthermore, the risk of CAD was found to be higher among employed patients than those who were unemployed. The results of a meta-analysis of 13 European cohort studies conducted between 1985 and 2006 revealed an increased risk of CVDs associated with increased workload and job strain [26]. Therefore, the type of job, including its time and duration, can be a risk factor for CVDs.

The results indicate that individuals with lower levels of education have a higher incidence of CAD than those with higher education levels. Similarly, previous studies have revealed that individuals with lower education are at a greater risk [27, 28], which supports the results of the present study. Individuals with low socioeconomic status and education tend to exhibit a higher prevalence of drug abuse, smoking, diabetes, hypertension, and hyperlipidemia as risk factors for CVDs (CVDs).

Based on the results, the prevalence of smoking and opium use is higher in patients with CAD compared to those without CAD. Alongside this, it has been reported that family history, smoking, and co-morbidities can increase the risk of CAD in patients [29]. Besides, there is a stronger correlation between CAD in men and the use of opium and smoking. In addition, higher prevalence of opium use in males results in different patterns of premature CAD [30] and coronary artery bypass grafting in patients [31]. Indeed, the results of our previous study indicated that opium consumption reduced the ejection fraction, which is a key variable for heart failure [32].

The results of a basic study indicated that the administration of opium decreased the serum levels of AST, ALT, total protein, total cholesterol, and TG while also resulting in an increase in urea and creatinine in diabetic rats [33]. The results of the PERSIAN cohort study revealed a positive correlation between opium consumption and lower levels of cholesterol and LDL. Additionally, a decrease in HDL levels was observed among opium abusers [34]. Further, several clinical studies have suggested that opium consumption does not have a significant effect on cholesterol, TG, LDL, or HDL-C [35, 36]. On the other hand, controversial evidence regarding opium use has been reported, including its potential effects on lowering lipid profile and FBS, reduction in left ventricular ejection fraction (LVEF), and enhancement of free radicals through the activation of lipid peroxidation [37]. The results of the aforementioned studies indicate that smoking and opium use are risk factors for CAD in patients.

Dyslipidemia and diabetes mellitus also have a higher prevalence in patients with premature CAD compared to non-CAD patients in this study. Metabolic syndrome has been linked to an elevated risk of CVD, according to reports. Atherogenic dyslipidemia is a significant modifiable risk factor in patients with CVD [38]. According to reports, type 2 diabetes is associated with a two to four times higher risk of CVD events and significantly raises mortality rates [39]. These results additionally support the findings of the current study.

The current study found that the prevalence of poison exposure as a lifestyle risk factor is greater among patients with CAD and premature CAD than among non-CAD patients. Organophosphorus pesticides (OPs) and household insecticides are widely used worldwide. The results of a cohort study revealed that patients who were acutely exposed to OPs had a higher incidence of arrhythmia, CAD, and CHF compared to patients who were not poisoned by OPs [40]. The adverse effects on heart rhythms may be caused by the suppression of the acetylcholinesterase (AChE) enzyme and the induction of oxidative stress in OPs. Exposure to pyrethroids, a type of insecticide, has been found to have a negative association with CVD and coronary heart disease in adults in the United States [41]. Furthermore, environmental exposure may play a crucial role in the development and severity of CVD. Likewise, air pollution and heavy metals can potentially exacerbate diseases by initiating or increasing pathophysiological processes. These processes include disruptions to carbohydrate and lipid metabolism, as well as impairments to vascular function that are commonly associated with CVD [42]. According to the findings of the studies above, air pollution, cigarette smoking, and opium use may increase the risk of CAD by increasing inflammation and oxidative stress.

Acceptance of stress has a higher prevalence, whereas anxiety has a lower prevalence in patients with CAD than in premature and non-CAD populations. Acceptance can be a valuable tool for alleviating stress.

It has been theorized that acceptance training is an essential element of mindfulness meditations to enhance health outcomes, affective reactivity, and stress levels [43]. Previous studies have indicated that certain psychiatric factors, such as chronic anxiety and daily stressors, have a negative impact on cardiovascular health [44, 45]. The results of a meta-analysis have also indicated that stress is implicated in the prognosis of CVD [46]. Short-term emotional stress has the potential to serve as a catalyst for cardiac events. Similarly, long-term stress, such as work-related stress and social isolation, has been found to be linked to CVD in individuals [45].

The results of the current study showed that the anxiety score was higher in non-CAD patients compared to CAD patients. This may be attributed to their “constructive worrying” and their early visits to physicians. The findings of a study also indicated that patients with generalized anxiety disorder have a capacity for constructive worrying and are more likely to seek help in response to less severe symptoms [47]. Furthermore, stress that is not “overwhelming” or “adequate acute stress” may enhance performance and can be beneficial in certain cases [46].

The results of the current study also showed that seasonal allergy was higher, but sexual activity was lower in non-CAD patients compared to premature CAD patients. Also, the prevalence of female gender and Anxiety in non-CAD patients was higher than premature CAD patients.

It has been reported that female patients with allergic rhinitis show significant higher levels of sensitivity to irritants and airway hyperresponsiveness than males. Furthermore, the female allergic subjects tended to have higher concentrations of substance P before and after non-specific challenges and difference between post allergen challenge was highly significant in female patients [48]. These data indicated that difference in seasonal allergy between premature CAD and non-CAD patients may be gender- related of patients.

In the other hands, seasonal allergies are a risk factor for psychiatric disorders, and association between seasonal allergies and eating disorders, substance use and mood disorders was also reported [49]. Sexual health is related to general health in both genders. Changes in lifestyle including, obesity, smoking and psychosocial factors (stress, depression and anxiety) can contribute and amplify the sexual dysfunction [50]. Weight loss is also associated with an improvement of function and quality of life [51]. According to the results of mentioned studies the significant difference in seasonal allergy and sexual activity might be attributed to the gender of patients.

In our study, the ANN predictors appear reasonable, as most of them are reportedly associated with CAD. In the current study, the most highly ranked and important risk factors for premature CAD and non-CAD patients include anxiety, acceptance, education, gender, and SBP. Low education level, mental stress, diabetes mellitus, hypertension, and obesity, as well as exposure to occupational and environmental risks, are the most significant risk factors for CAD [52]. The epidemiologic studies also suggest that individuals with psychological stress are at an increased cardiovascular risk [53].

In addition, Deep Neural Network techniques were used to create a model for predicting the risk of CVD in type 2 diabetes mellitus patients. The top five predictors in the CVD risk prediction model were BMI, anxiety, depression, total cholesterol, and SBP [54]. Additionally, according to the ANN model, depression, anxiety, and BMI are identified as the three most significant predictive factors for heart attacks. The predictors selected by the ANN in our study are consistent with previous reports [55]. The current study has limitations in terms of design and analysis, specifically related to issues such as reverse causality, incidence-prevalence bias, and unmeasured confounding.

## Conclusions

This article introduced an ANN-based model of CAD risk prediction. The proposed model enhances CAD risk assessment and decision support for appropriate treatment. While gender, education, smoking, opium use, history of dyslipidemia, and diabetes have been identified as potential risk factors for CAD, it is not clear if they can be accurately predicted using ANN for CAD risk assessment. There is no association found between a history of high dyslipidemia, diabetes mellitus, and fasting blood sugar in relation to CAD. However, it has been determined that anxiety, acceptance, and gender are the most significant factors contributing to CAD. Although more progress has been made in understanding the contribution of psychological disorders to CAD, further clinical studies are needed to elucidate these mechanisms.

## Data Availability

All data generated or analyzed during this study are included in the article.

## Abbreviations

AChE: Acetylcholinesterase
ANN: Artificial neural network
CABG: Coronary artery bypass grafting
CAD: Coronary artery disease
Chol: Cholesterol
CVDs: Cardiovascular diseases
DNN: Deep Neural Network
FBS: Fasting blood sugar
FFQ: Food Frequency Questionnaire
GAD: Generalized anxiety disorder
HADS: Anxiety and Depression Scale
HDL-c: High-density lipoprotein cholesterol
I-PAD: Iran-premature coronary artery disease
IPAQ: International Physical Activity Questionnaire
LDLc: Low-density lipoprotein cholesterol
LM: Left main
NN: Neural network
PCI: Percutaneous coronary intervention
PSQI: Sleep quality using the Pittsburgh Sleep Quality Index
SBP: Systolic Blood Pressure
T2DM: Type 2 diabetes mellitus
TG: Triglyceride
